# Profiling the Countermovement Jump Characteristics of Basketball Players across Competitive Levels and Playing Positions

**DOI:** 10.5114/jhk/196138

**Published:** 2025-04-30

**Authors:** Carlos Suárez-Balsera, Davide Ferioli, Elena Marín-Cascales, Vincenzo Rago, Konstantinos Spyrou, Antonio Martínez-Serrano, Debora Di Mauro, José Manuel Marín, Pedro E. Alcaraz, Tomás T. Freitas

**Affiliations:** 1 UCAM Research Center for High Performance Sport, UCAM Catholic University of Murcia, Murcia, Spain.; 2 Department of Biomedical Sciences, Dental Sciences, and Morpho-Functional Imaging, University of Messina, Messina, Italy.; 3 Faculty of Sport, Catholic University of Murcia, Murcia, Spain.; 4 SCS—Strength & Conditioning Society, Murcia, Spain.; 5 Faculty of Health and Sports Sciences, Universidade Europeia, Lisbon, Portugal.; 6 UCAM CB, UCAM Catholic University of Murcia, Murcia, Spain.; 7 NAR—Nucleus of High Performance in Sport, Sao Paulo, Brazil.

**Keywords:** vertical jump, team sport, neuromuscular, CMJ, playing role

## Abstract

This study aimed to examine different force-time derived variables of the countermovement jump (CMJ) in adult, male basketball players of different competitive levels and playing positions. At the beginning of the pre-season, 78 players from two competitive levels (professional and semi-professional) completed a CMJ test on a force plate. Linear mixed models were used to compare a total of 21 metrics across competitive levels and playing positions. Professional players jumped significantly higher (p = 0.022) than semi-professionals. No differences were observed in the variables related to the eccentric phase. Four concentric phase variables (i.e., absolute peak force [p < 0.001], absolute and relative peak power [p < 0.001 and p = 0.037, respectively] and peak velocity [p = 0.036]) were superior in professional in comparison to semi-professional players. Regarding the playing position, centers presented significantly larger deceleration phase duration than guards (p = 0.042). Centers reported higher absolute eccentric peak force than guards (p = 0.013). In the concentric phase, centers reported larger concentric duration than forwards (p = 0.011) and guards (p = 0.002). Guards produced lower absolute peak force compared to centers (p = 0.004) and forwards (p = 0.025). Furthermore, centers were characterized by lower levels of relative peak force (p = 0.013), but higher levels of absolute peak power compared to guards (p = 0.003). In conclusion, jump height, and only four concentric-phase metrics were found to discriminate between competitive levels. Additionally, the eccentric deceleration phase duration, absolute eccentric peak force, concentric duration, absolute and relative peak force and absolute peak power discriminated between playing positions. Practitioners should conduct thorough CMJ analyses and consider these metrics from a performance and talent identification perspective.

## Introduction

Basketball is an intermittent team-sport characterized by repeated transitions between offensive and defensive actions that involve high-intensity efforts interspersed with low-intensity activities (Petway et al., 2020). To succeed, players should not only display great tactical and technical mastery, but also be proficient at a wide variety of physical and motor skills (e.g., linear and multidirectional acceleration and deceleration, shuffling, jumping, etc.) (Barrera-Dominguez et al., 2024; Lazić et al., 2022; Petway et al., 2020; Stojanovic et al., 2018). Among these, the vertical jump is conceivably one of the most significant, as crucial game actions such as shooting, rebounding and shot-blocking all involve jumping efforts. Performance in the vertical jump is determined by a complex interaction between several factors, including maximum force production by the involved muscle groups, the rate at which this force is developed and neuromuscular coordination of the lower and upper limbs (McLellan et al., 2011). Thus, vertical jump performance analysis, mainly through the countermovement jump (CMJ) test, has long been a topic of interest for basketball practitioners and academics (Ferioli et al., 2018; Metaxas et al., 2009).

Of note, higher running demands have been reported in professional than semi-professional level players. Indeed, professional players execute greater moderate-high intensity intermittent actions (e.g., accelerations, decelerations, and changes of direction) and sustain more physical demands than semi-professional athletes (Ferioli et al., 2020; Scalan et al., 2015). Still, no differences have been observed when considering the jumping demands, probably because this specific skill is a basic requirement at all levels (Ferioli et al., 2020). Additionally, professional players present superior stature, muscle mass and a greater ability to sustain high-intensity intermittent efforts and repeated changes of direction (Ferioli et al., 2018, 2019). However, studies comparing strength and the vertical jump in players of different competitive levels have reported conflictive results (Abdelkrim et al., 2010; Ferioli et al., 2018, 2020; Koklu et al., 2011). Thus, further research and more solid evidence is still warranted.

Although superior relative force/power variables can allow players to obtain advantages in the basketball court, Ferioli et al. (2018) found only small differences in CMJ height, relative peak power and peak force (i.e., normalized to body mass) when comparing Division I, II, and III male adult players. Conversely, those authors identified meaningful differences in the absolute peak force and peak power, suggesting that these variables could be used to discriminate between players competing at different levels (Ferioli et al., 2018). Nevertheless, it is worth noting that Ferioli et al. (2018) analyzed exclusively jump height, power and force metrics. A singular study by Cabarkapa et al. (2023) has performed a more complete analysis of the different phases (i.e., eccentric and concentric) of the CMJ during the in-season period (comparing starters to non-starters) and found no statistically significant differences in any of the 19 CMJ variables analyzed as well as in anthropometric characteristics (i.e., stature, body mass) and players’ age. Thus, to date, no study has investigated the eccentric and concentric phase-specific metrics of the CMJ (which is one of the most used tests in basketball (Dias et al., 2011)) comparing between competitive levels. This information is lacking in literature and may be of interest for practitioners and coaching staff for talent identification and player development purposes. Furthermore, since in other sports in which the vertical jump is not as crucial as basketball for competitive success (i.e., futsal), selected variables related to the eccentric and concentric phase of the CMJ have already been shown to differentiate professional from semi-professional players (Spyrou et al., 2024), thus differences may also be expected among adult basketball players competing at different levels.

Besides the competitive status, positional roles are also relevant, based on the different match-play demands (Ferioli et al., 2020; Stojanovic et al., 2018) and anthropometric features reported (e.g., centers are typically heavier and taller than both forwards and guards (Ferioli et al., 2018; Vaquera et al., 2015)). Regarding vertical jump performance, CMJ height has been reported to be only slightly greater in guards (when compared to forwards and centers) (Ferioli et al., 2018; Koklu et al., 2011); thus, other metrics such as relative and absolute peak power have been suggested as possibly superior discriminative variables (Ferioli et al., 2018; Metaxas et al., 2009; Ostojic et al., 2006). Recently, Cabarkapa et al. (2023) performed a thorough phase-specific analysis of the CMJ in basketball players, reporting differences between playing positions in different outcomes (i.e., braking impulse, mean force and mean power in the eccentric phase, concentric duration, impulse, relative mean and peak force, and relative mean and peak power in the concentric phase). However, despite the novel and interesting results, special attention and caution should be taken when analyzing and interpreting the evidence found by Cabarkapa et al. (2023) as those authors assessed a relatively limited number of players per position (e.g., only 5 centers). Hence, despite differences in jumping ability may be expected between playing positions based on previous research (Cabarkapa et al., 2023), more evidence is still needed, particularly with greater sample sizes.

Therefore, the aim of this study was to explore whether there were differences between a myriad of eccentric and concentric phase-specific CMJ force-time metrics according to two different competitive levels (i.e., first and fourth division in Spain) and three different playing positions (i.e., guards, forwards and centers) in men's basketball. It was hypothesized that: 1) professional players would outperform their semi-professional counterparts in the majority of the variables analyzed; 2) centers would display greater absolute, but not relative (i.e., normalized to body mass), force and power output than forwards and guards; and 3) guards would jump higher and reach greater eccentric and concentric peak velocities than the other positions.

## Methods

### Participants

A convenience sample of 78 adult male players competing in the Spanish first division (i.e., *Liga ACB*) (professional players, n = 39; age [estimated marginal means and 95% confidence intervals, CI]: 27.0 ± 3.6 years, stature: 198 ± 8.9 cm, body mass: 95.0 ± 11.8 kg) and fourth division (i.e., *Liga EBA*) (semi-professional players, n = 39; age: 19.3 ± 1.7 years, stature: 197 ± 8.0 cm, body mass: 88.5 ± 10.2 kg) were recruited from the same elite basketball organization encompassing a professional and a semi-professional team throughout the 2014–2021 seasons. The sample comprised players from different playing positions: guards (n = 25; age: 23.2 ± 5.2 years, stature: 188 ± 4.8 cm, body mass: 82.5 ± 9.1 kg), forwards (n = 37; age: 23.1 ± 5.1 years, stature: 198 ± 6.3 cm, body mass: 93.0 ± 8.1 kg), and centers (n = 16; age: 22.9 ± 3.5 years, stature: 206 ± 3.4 cm, body mass: 102.3 ± 10.8 kg) that were equally represented in each group: 13, 19 and 7 professional guards, forwards and centers, respectively; and 12, 18, and 9 semi-professional guards, forwards and centers, respectively. Players reported no injury at the moment of testing. By signing a professional contract with the club, all players provided individual written informed consent for data collection and study participation. The study was approved by the Institutional Review Board of the Catholic University of Murcia (protocol code: CE072008; approval date: 20 July 2020) in accordance with the Helsinki Declaration.

### Design

This observational study was conducted over an 8-year spam, across the 2014–2021 seasons, with players being assessed during the month of August (coinciding with the first weeks of the preparation period) in the morning (e.g., 09:00–13:00). Across the 8-year period, each player was tested in the season or seasons in which he was a member of the basketball organization, resulting in a range of 1 to 7 measurements per player (i.e., no player was assessed 8 times). During the testing day, a battery of tests was performed, in the following order: anthropometric measurements, vertical jump, sprint and change of direction ability tests. However, for the present study, only anthropometric and vertical jump data were considered. To avoid potential confounding fatigue effects, players did not perform any high-intensity activity the day prior to the test. All players were well familiarized with the tests performed in this study. Moreover, across the 8-season period considered for the study, all assessments were conducted at the same facilities, with the same testing equipment administered by experienced researchers and supervised by the same performance staff member.

### Procedures

Players performed a standardized 15-min warm-up, encompassing dynamic stretching, mobility exercises, lower-body exercises (bodyweight squats, lunges and lateral lunges, and band-resisted adductors and abductors exercises), running-based exercises (jogging, multidirectional displacements and sprints) and three submaximal jumps (Zhao et al., 2023). No static stretching was allowed during the warm-up. Within 5 min from the end of the warm-up, players performed a set of three CMJs on a portable force plate (Kistler 9286BA, Kistler Group, Winterthur, Switzerland) separated by 45–60 s of passive rest. During the execution of each CMJ, players were required to start from a standing position with the hands placed on the hips and to perform a quick downward movement reaching approximately 90º of knee flexion, followed by a quick upward movement with the goal to jump as high as possible. Multiple variables (described in Table 1) were recorded during the CMJs and analyzed using specific software (ForceDecks, VALD Performance, Brisbane, Australia). Average values (out of the three CMJs) of the analyzed metrics were used for the statistical analysis.

**Table 1:** Brief description of the different phases of the countermovement jumps and of all the related variables.

Phases	Description
Eccentric	From start of movement until velocity = 0 m•s^−1^
Braking	Eccentric subphase: from point of minimum force until velocity = 0 m•s^−1^
Deceleration	Eccentric subphase: from peak negative velocity to 0 m•s^−1^
Concentric	From positive velocity from = m•s^−1^ until takeoff
Flight	From vertical force drops below 20 N until vertical force returns above 20 N
**Variable (units)**	**Description**
Jump height Imp-Mom (cm)	Jump height calculated from the velocity of the center of mass at the instant of take-off and body mass
Flight time:Contraction time	Ratio of flight time to contraction time
Reactive strength index modified (m‧s^−1^)	Jump height divided by contraction time
Eccentric braking phase duration (s)	Time period of the eccentric braking phase
Eccentric braking phase duration: contraction time	Ratio of braking phase duration to contraction time
Countermovement depth (cm)	Maximum displacement between start of movement to take-off
Eccentric deceleration phase duration (s)	Time period of the eccentric deceleration phase
Eccentric duration (ms)	Time period of the eccentric phase
Eccentric absolute peak force (N)	Maximum vertical force during the eccentric phase
Eccentric relative peak force (N‧kg^−1^)	Maximum force achieved during the eccentric phase relative to the athlete´s weight
Eccentric absolute peak power (W)	Maximum power during the eccentric phase
Eccentric relative peak power (W‧kg^−1^)	Maximum power achieved during the eccentric phase relative to the athlete´s weight
Eccentric peak velocity (m‧s^−1^)	Maximum velocity during the eccentric phase
Concentric duration (ms)	Time period of the concentric phase
Concentric absolute peak force (N)	Maximum vertical force during the concentric phase
Concentric relative peak force (N‧kg^−1^)	Maximum vertical force achieved during the concentric phase relative to the athlete´s weight
Concentric absolute peak power (W)	Maximum power achieved during the concentric phase
Concentric relative peak power (W‧kg^−1^)	Maximum power achieved during the concentric phase relative to the athlete´s weight
Concentric peak velocity (m‧s^−1^)	Maximum velocity during the concentric phase
Contraction time (ms)	Time period from start of movement to take-off
Concentric velocity at peak power (m‧s^−1^)	Velocity of the center of mass at the point when peak power between start of movement and take-off occurs

### Statistical Analysis

Before running linear mixed models, boxplots and histograms were used to identify and exclude potentially influential data points with no outliers being detected. Following this analysis, residual plots were visually inspected to determine deviations from homoscedasticity or normality. All assumptions were met, and the normality of the residuals was also assessed using the Kolmogorov-Smirnov and the Shapiro-Wilk tests. Linear mixed models were constructed to examine differences in CMJ variables according to the competitive level and the playing position, accounting for individual repeated measures. In all linear mixed models, the competitive level (two levels) and the playing position (three levels) were used as fixed effects and the player as a random effect with a random intercept and a fixed slope. Pairwise comparisons were performed using post-hoc tests with the Bonferroni correction factor applied to the playing position analyses. According to previous research (Rampinini et al., 2022), the *t* statistics from the mixed model were converted into Cohen’s *d* effect sizes (ES) and the associated 95% CI using the compute.es package (tes (t, n1, n2, level = 95) in R statistical software (version 4.1.3) (R Core Team. R: A language and environment for statistical computing; R Foundation for Statistical Computing, Vienna, Austria, 2019). ESs were interpreted as follows: < 0.2, trivial; 0.20–0.59, small; 0.60–1.19, moderate; 1.2–1.99, large and ≥ 2.0, very large (Hopkins et al., 2009). An alpha level of *p* < 0.05 was set a priori for statistical significance. Test-retest reliability of CMJ variables was determined using the intraclass correlation coefficient (Table 2) and classified as follows: < 0.5, poor reliability; 0.5–0.75, moderate reliability; 0.75–0.9, good reliability; and > 0.90, excellent reliability (Koo and Li., 2016). All data were analyzed using Jamovi software (version 1.8, 2021, retrieved from https://www.jamovi.org).

**Table 2:** Intraclass correlation coefficients with the respective 95% confidence intervals and interpretation of all the variables assessed.

Variable (units)	ICC (95%CI)	Interpretation
***Performance variables***		
Countermovement jump height Imp-Mom (cm)	0.922 (0.764–0.975)	Excellent
Flight time:Contraction time	0.918 (0.755–0.974)	Excellent
Reactive strength index modified (m‧s^−1^)	0.953 (0.855–0.986)	Excellent
***Eccentric (“downward”) phase***		
Eccentric braking phase duration (s)	0.753 (0.368–0.917)	Good
Eccentric braking phase duration: contraction time	0.592 (0.087–0.855)	Moderate
Countermovement depth (cm)	0.756 (0.373–0.918)	Good
Eccentric deceleration phase duration (s)	0.934 (0.800–0.979)	Excellent
Eccentric duration (ms)	0.846 (0.570–0.950)	Good
Eccentric absolute peak force (N)	0.970 (0.905–0.991)	Excellent
Eccentric relative peak force (N‧kg^−1^)	0.948 (0.840–0.984)	Excellent
Eccentric absolute peak power (W)	0.765 (0.392–0.922)	Good
Eccentric relative peak power (W‧kg^−1^)	0.724 (0.311–0.907)	Moderate
Eccentric peak velocity (m‧s^−1^)	0.791 (0.441–0.931)	Good
***Concentric (“upward”) phase***		
Concentric duration (ms)	0.906 (0.722–0.970)	Excellent
Concentric absolute peak force (N)	0.979 (0.933–0.994)	Excellent
Concentric relative peak force (N‧kg^−1^)	0.960 (0.876–0.988)	Excellent
Concentric absolute peak power (W)	0.961 (0.878–0.988)	Excellent
Concentric relative peak power (W‧kg^−1^)	0.871 (0.631–0.959)	Good
Concentric peak velocity (m‧s^−1^)	0.924 (0.771–0.976)	Excellent
Contraction time (ms)	0.873 (0.637–0.959)	Good
Concentric velocity at peak power (m‧s^−1^)	0.920 (0.759–0.975)	Excellent

*Abbreviations: CI: Confidence Interval; ICC: Intraclass Correlation Coefficient*

## Results

The results are reported as estimated marginal means with the 95% CI. Descriptive data and statistical analyses for CMJ variables according to the competitive level are presented in Table 3. Professional players jumped significantly higher than semi-professional players (ES = −0.48, *p* = 0.022). Trivial-to-small differences (all *p* > 0.05) were observed in CMJ variables measured during the eccentric phase according to the competitive level. During the concentric phase, professional players generated greater absolute peak force (ES = −0.96, *p* < 0.001), absolute peak power (ES = −1.03, *p* < 0.001) and relative peak power (ES = −0.44, *p* = 0.037), and achieved greater peak velocity (ES = −0.44, *p* = 0.036) compared to semi-professional players.

**Table 3:** Comparison of the countermovement jump variables according to the competitive level.

Variable (units)	EMMeans (95%CI)		ES (95%CI)	*p* value
	PRO	SEMI-PRO		
***Performance variables***				
Countermovement jump height Imp- Mom (cm)	39.2 (37.3; 41.1)	36.0 (34.0; 37.9)	-0.48^s^ (-0.89; -0.07)	**0.022**
Flight time:Contraction time	0.780 (0.741; 0.818)	0.743 (0.704; 0.782)	-0.27^s^ (-0.68; 0.13)	0.187
Reactive strength index modified (m-s^-1^)	0.545 (0.508; 0.582)	0.501 (0.464; 0.538)	-0.34^s^ (-0.75; 0.06)	0.099
***Eccentric (“downward” phase***				
Eccentric braking phase duration (s)	0.294 (0.279; 0.310)	0.296 (0.281; 0.312)	0.04^T^ (-0.36; 0.44)	0.844
Eccentric braking phase duration: contraction time	39.8 (38.5; 41.0)	39.4 (38.1; 40.6)	-0.09^T^ (-0.50; 0.31)	0.657
Countermovement depth (cm)	-30.3 (-32.4; -28.3)	-32.6 (-34.6; -30.7)	-0.33^s^ (-0.74; 0.07)	0.110
Eccentric deceleration phase duration (s)	0.168 (0.158; 0.178)	0.165 (0.155; 0.175)	-0.10^T^ (-0.51; 0.30)	0.623
Eccentric duration (ms)	482 (462; 502)	482 (461; 502)	-0.01^T^ (-0.41; 0.40)	0.969
Eccentric absolute peak force (N)	2187 (2085; 2289)	2046 (1944; 2148)	-0.40^s^ (-0.81; 0.01)	0.056
Eccentric relative peak force (N-kg^-1^)	22.7 (21.7; 23.7)	23.2 (22.2; 24.2)	0.15^T^ (-0.26; 0.55)	0.471
Eccentric absolute peak power (W)	1718 (1565; 1871)	1718 (1565; 1871)	0.04^T^ (-0.37; 0.44)	0.999
Eccentric relative peak power (W-kg^-1^)	17.9 (16.2; 19.5)	19.5 (17.8; 21.1)	0.28^s^ (-0.13; 0.69)	0.178
Eccentric peak velocity (m-s^-1^)	-1.25 (-1.32; -1.17)	-1.33 (-1.40; -1.26)	-0.34^s^ (-0.74; 0.07)	0.108
***Concentric (“upward”) phase***				
Concentric duration (ms)	257 (246; 268)	269 (257; 280)	0.30^s^ (-0.11; 0.70)	0.156
Concentric absolute peak force (N)	2471 (2383; 2558)	2182 (2094; 2270)	-0.96^M^ (-1.38; -0.53)	**<0.001**
Concentric relative peak force (N-kg^-1^)	25.7 (24.9; 26.5)	24.8 (24.0; 25.6)	-0.33^s^ (-0.74; 0.08)	0.115
Concentric absolute peak power (W)	5520 (5297; 5743)	4718 (4487; 4949)	-1.03M (-1.46; -0.60)	**<0.001**
Concentric relative peak power (W-kg^-1^)	57.2 (54.9; 59.5)	53.8 (51.5; 56.1)	-0.44^s^ (-0.85; -0.03)	**0.037**
Concentric peak velocity (m-s^-1^)	2.89 (2.82; 2.95)	2.79 (2.73; 2.86)	-0.44^s^ (-0.85; -0.03)	**0.036**
Contraction time (ms)	739 (711; 766)	750 (722; 779)	0.12^T^ (-0.29; 0.52)	0.566
Concentric velocity at peak power (m-s^-1^)	2.53 (2.47; 2.59)	2.48 (2.42; 2.54)	-0.26^s^ (-0.67; 0.15)	0.210

Abbreviations: CI = Confidence Interval; EMMeans = Estimated Marginal Means; ES = effect size; PRO = professional players competing in the Spanish first division; SEMI-PRO = semi-professional players competing in the Spanish fourth division. Notes: Bolden p value indicates statistically significant difference at p < 0.05. ^T^ trivial effect size; ^S^ small effect size; ^M^ moderate effect size

Descriptive data and statistical analyses for CMJ variables according to the playing position are presented in Table 4. During the eccentric phase, centers were observed to be significantly slower in execution (i.e., deceleration phase duration) compared to guards (ES = 0.73, *p* = 0.042), while guards produced lower absolute peak force compared to centers (ES = 0.85, *p* = 0.013). During the concentric phase, forwards (ES = 0.80, *p* = 0.011) and guards (ES = 1.03, *p* = 0.002) were observed to be significantly faster in execution compared to centers. Guards produced lower absolute peak force compared to centers (ES = 0.96, *p* = 0.004) and forwards (ES = 0.65, *p* = 0.025). Furthermore, centers were characterized by lower levels of relative peak force (ES = −0.85; *p* = 0.013), but higher levels of absolute peak power (ES = 1.00, *p* = 0.003) compared to guards.

**Table 4:** Comparison of the countermovement jump variables according to the playing position.

		EMMeans (95%CI)		Main effect *p* value	Centers vs. Forwards		Centers vs. Guards		Forwards vs. Guards	
Dependent variable (units)	Guards	Forwards	Centers		ES (95%CI)	*p* value	ES (95%CI)	*p* value	ES (95%CI)	*p* value
***Performance variables***										
Countermovement jump height Imp- Mom (cm)	38.9 (36.6; 41.2)	37.2 (35.3; 39.1)	36.6 (33.8; 39.5)	0.393	-0.08^T^ (-0.60; 0.43)	1.000	-0.35^s^ (-0.92; 0.22)	0.672	-0.27^s^ (-0.74; 0.20)	0.782
Flight time:Contraction time	0.794 (0.748; 0.840)	0.764 (0.727; 0.802)	0.726 (0.669; 0.782)	0.183	-0.30^s^ (-0.82; 0.22)	0.792	-0.54^s^ (-1.11; 0.04)^S^	0.203	-0.24^s^ (-0.71; 0.23)	0.982
Reactive strength index modified (m-s^-1^)	0.558 (0.514; 0.603)	0.521 (0.484; 0.557)	0.491 (0.436; 0.545)	0.151	-0.24^s^ (-0.76; 0.28)	1.000	-0.55^s^ (-1.13; 0.02)	0.177	-0.31^s^ (-0.78; 0.16)	0.590
***Eccentric (“downward”) phase***										
Eccentric braking phase duration (s)	0.286 (0.267; 0.304)	0.287 (0.272; 0.302)	0.313 (0.291; 0.336)	0.114	0.51^s^ (-0.01; 1.03)	0.174	0.55^s^ (-0.03; 1.12)	0.189	0.03^T^ (-0.44; 0.50)	1.000
Eccentric braking phase duration:Contraction time	39.3 (37.8; 40.0)	49.3 (38.1; 40.5)	40.1 (38.3; 42.0)	0.708	0.21^s^ (-0.31; 0.73)	1.000	0.20^s^ (-0.37; 0.77)	1.000	-0.01^T^ (-0.48; 0.46)	1.000
Countermovement depth (cm)	-31.1 (-33.5; -28.8)	-30.4 (-32.4; -28.5)	-32.9 (-35.9; -30.0)	0.370	-0.37^s^ (-0.89; 0.15)	0.480	-0.27^s^ (-0.84; 0.30)	1.000	0.11^T^ (-0.36; 0.58)	1.000
Eccentric deceleration phase duration (s)	0.158 (0.146; 0.169)	0.161 (0.151; 0.170)	0.181 (0.167; 0.195)	**0.030**	0.62M (0.09; 1.15)	0.064	0.73M (0.14; 1.31)	**0.042**	0.09^T^ (-0.38; 0.56)	1.000
Eccentric duration (m-s^-1^)	479 (455; 504)	475 (455; 495)	491 (462; 521)	0.664	0.24^s^ (-0.28; 0.75)	1.000	0.18^T^ (-0.39; 0.75)	1.000	-0.06^T^ (-0.53; 0.41)	1.000
Eccentric absolute peak force (N)	1959 (1837; 2080)	2148 (2048; 2247)	2244 (2095; 2393)	**0.010**	0.28^s^ (-0.24; 0.80)	0.862	0.85M (0.26; 1.44)	**0.013**	0.57^s^ (0.09; 1.05)	0.058
Eccentric relative peak force (N-kg^-1^)	23.7 (22.5; 24.8)	23.2 (22.2; 24.1)	22.0 (20.6; 23.5)	0.224	-0.34^s^ (-0.86; 0.18)	0.594	-0.50^s^ (-1.07; 0.07)	0.264	-0.15^T^ (-0.62; 0.32)	1.000
Eccentric absolute peak power (W)	1576 (1393; 1758)	1770 (1622; 1919)	1808 (1585; 2031)	0.176	0.07^T^ (-0.44; 0.59)	1.000	0.46^s^ (-0.11; 1.04)	0.340	0.39^s^ (-0.08; 0.87)	0.312
Eccentric relative peak power (W-kg^-1^)	19.1 (17.1; 21.1)	19.1 (17.5; 20.8)	17.7 (15.3; 20.2)	0.606	-0.25^s^ (-0.77; 0.27)	1.000	-0.25^s^ (-0.82; 0.32)	1.000	0.00^T^ (-0.47; 0.47)	1.000
Eccentric peak velocity (m-s^-1^)	-1.29 (-1.38; -1.21)	-1.29 (-1.36; -1.21)	-1.29 (-1.39; -1.18)	0.985	-0.01^T^ (-0.53;0.51)	1.000	0.03^T^ (-0.54; 0.60)	1.000	0.04^T^ (-0.43; 0.51)	1.000
***Concentric (“-upward”) phase***										
Concentric duration ^(ms)^	248 (234; 261)	256 (245; 267)	286 (269; 302)	**0.002**	0.80M (0.26; 1.33)	**0.011**	1.03M (0.42; 1.63)	**0.002**	0.22^s^ (-0.26; 0.69)	1.000
Concentric absolute peak force (N)	2172 (2068; 2277)	2357 (2271; 2442)	2450 (2322; 2578)	**0.003**	0.32^s^ (-0.20; 0.83)	0.699	0.96M (0.37; 1.56)	**0.004**	0.65M (0.17; 1.13)	**0.025**
Concentric relative peak force (N-kg^-1^)	26.3 (25.3; 27.3)	25.4 (24.7; 26.2)	24.0 (22.8; 25.2)	**0.016**	-0.53^s^ (-1.05; -0.01)	0.147	-0.85^M^ (-1.44; -0.26)	**0.013** )	-0.32^s^ (-0.79; 0.15)	0.564
Concentric absolute peak power (W)	4763 (4489; 5036)	5082 (4861; 5304)	5511 (5183; 5839)	**0.004**	0.57^s^ (0.04; 1.09)	0.108	1.00M (0.40; 1.60)	**0.003**	0.43^s^ (-0.05; 0.90)	0.231
Concentric relative peak power (W-kg^-1^)	57.7 (55.0;60.5)	54.9 (52.6; 57.1)	53.9 (50.6; 57.3)	0.151	-0.12^T^ (-0.64; 0.40)	1.000	-0.51^s^ (-1.08; 0.07)	0.249	-0.39^s^ (-0.87; 0.08)	0.323
Concentric peak velocity (m-s^-1^)	2.88 (2.80; 2.95)	2.82 (2.76; 2.88)	2.82 (2.73; 2.91)	0.484	-0.01^T^ (-0.53; 0.51)	1.000	-0.27^s^ (-0.84; 0.29)	1.000	-0.27^s^ (-0.74; 0.20)	0.805
Contraction time (ms)	727 (693; 761)	730 (703; 757)	777 (736; 817)	0.119	0.50^s^ (-0.03; 1.02)	0.191	0.54^s^ (-0.04; 1.12)	0.197	0.03^T^ (-0.44; 0.50)	1.000
Concentric velocity at peak power (m-s^-1^)	2.53 (2.46; 2.60)	2.49 (2.44; 2.55)	2.49 (2.41; 2.58)	0.695	0.00^T^ (-0.51; 0.52)	1.000	-0.19T (-0.76; 0.38)	1.000	-0.19^T^ (-0.66; 0.28)	1.000

Abbreviations: CI = Confidence Interval; EMMeans = Estimated Marginal Means; ES = effect size. Notes: Bolden p value indicates statistically significant difference at p < 0.05.T trivial effect size;S small effect size; M moderate effect size

## Discussion

This study analyzed a myriad of variables from the eccentric and concentric phases of the CMJ, examining the differences between professional and semi-professional male basketball players and accounting for different playing positions (i.e., guards, forwards and centers). The main findings were that: 1) professional players jumped higher and, during the concentric phase, produced greater absolute peak force and absolute and relative peak power, and achieved higher peak velocity compared to semi-professional players; 2) the eccentric phase metrics analyzed did not differ when comparing professional and semi-professional players; 3) centers displayed longer eccentric deceleration phase duration and greater absolute peak force compared to guards; 4) centers presented higher concentric duration and absolute concentric peak force and power, but lower concentric relative peak force than guards; 5) forwards displayed shorter concentric duration compared to centers and higher concentric absolute peak force compared to guards.

Numerous studies (Abdelkrim et al., 2010; Ferioli et al., 2018, 2019; Köklü et al., 2011) have compared the physical characteristics of basketball players from different competitive levels; still, the investigations that have analyzed CMJ height, power and force metrics have shown contradicting results. In this sense, contrary to Ferioli et al. (2018), but in line with Köklü et al. (2011), professional players displayed greater CMJ height than their semi-professional counterparts. However, only small differences were found, which indicates that while this metric should be considered by practitioners, it might not be the main discriminating variable. Furthermore, CMJ height values of professional players measured in this study (~39 cm) were lower than those of professional players (~47 cm) competing in the Italian first division (i.e., Serie A) recorded at the same time-point of the season (i.e., beginning of the preparation period) (Ferioli et al., 2020). The precise causes for the discrepancies in the reported values are difficult to determine. However, it could be argued that the differences might, at least in part, be attributable to the use of different force plates (Kistler 9286BA vs. Kistler Quattro jump) and, more specifically, to the dissimilar calculation methods adopted by the proprietary software (VALD performance software vs. Quattro jump software) to determine CMJ height (Linthorne, 2001). Interestingly, the values obtained here by professional and semi-professional players in all metrics measured during the eccentric phase of the CMJ were similar, suggesting that these variables, despite their importance, do not discriminate between players from different competitive levels. Accordingly, Cabarkapa et al. (2023) also found these metrics were not relevant to discriminate between starters and non-starters, thus further strengthening the notion that eccentric phase variables seem not to be the most determinant when assessing CMJ performance in basketball players. On the other hand, some metrics evaluated during the concentric phase of the CMJ were found to discriminate between players according to their competitive level. In line with previous research, the absolute peak force (Ferioli et al., 2018) and the absolute peak power (Abdelkrim et al., 2010; Ferioli et al., 2018) were moderately greater among higher level players, likely as a consequence of the greater muscular mass of professional compared to semi- professional players. Also, significant differences in relative peak power and peak velocity were found in favor of professional players, which may have probably contributed (along with the non-significant differences found among duration-related variables) to greater CMJ height in this group of athletes (Krzyszkowski et al., 2020). Thus, the present data hint that greater focus should be placed on developing absolute levels of power and strength in talented basketball players, as these qualities might be advantageous to compete at a higher level.

Regarding the playing position, guards achieved jumping heights ~4% and ~6% higher than forwards and centers, respectively. However, in line with a recent study by Cabarkapa et al. (2023), the statistical analyses revealed no significant differences among playing positions. This result does not support previous studies on the topic highlighting the playing position discriminant validity of this metric (Berg et al., 1994; Boone and Burgois, 2013; Vaquera et al., 2015). Noteworthy, in contrast with recent literature (Cabarkapa et al., 2023), the eccentric deceleration phase duration was longer in centers when compared to guards. Moreover, the eccentric absolute peak force was lower in guards in comparison with centers which also contradicts the findings by Cabarkapa et al. (2023) who reported no differences among playing positions. In the same way, those authors observed greater values of eccentric mean force and power in centers than in both forwards and guards (Cabarkapa et al., 2023). Therefore, the movement in the eccentric phase in centers appears to be slower and requires greater force production, conceivably because of the unique anthropometric characteristics of these players (e.g., centers are heavier and taller and produce slower but more forceful movements). In what concerns the concentric phase, the present results support previous literature in several aspects: 1) the concentric phase duration was shorter in guards and forwards in comparison with centers (Cabarkapa et al., 2023); 2) the concentric relative peak force was greater in guards in comparison with centers (Cabarkapa et al., 2023; Ferioli et al., 2018); and 3) centers developed greater absolute peak power than guards (Boone and Burgois, 2013; Ferioli et al., 2018; Sallet et al., 2005; Vaquera et al., 2015). However, there are also findings herein that contrast with other investigations (Cabarkapa, 2023). For example, the current study reported greater concentric absolute peak force in centers and forwards in comparison to guards, whereas Cabarkapa et al. (2023) found no meaningful differences. Conflictive results were also found regarding the absolute and relative concentric peak power. While in the present study differences among positions were observed only in absolute values (i.e., centers > guards), Cabarkapa et al. (2023) reported significant differences solely in relative values (i.e., guards > forwards > centers). Altogether, these results seem to confirm that centers can reach higher absolute peak force and peak power values, although when force and power values are normalized to body mass, lighter players (e.g., guards) tend to display superior outcomes. The inconsistencies found among studies in some of the metrics previously discussed might be attributed to potential disparities in physical demands and players’ profiles of different countries (e.g., Spanish vs. Adriatic Leagues). Furthermore, it is noteworthy to consider that the present study encompassed a larger sample of players assessed across multiple seasons, thus enhancing its statistical power and ecological validity. From an applied perspective, the results of the present study reinforce the importance of conducting an in-depth kinetic analysis of the CMJ using force plates (Nishiumi and Hirose, 2024) to more accurately profile and assess basketball players. Still, more evidence is required regarding the differences between playing positions to provide researchers and practitioners with clearer and robust conclusions.

Some limitations of the present study must be addressed. Firstly, semi-professional players (i.e., fourth division) were, on average, younger than their professional counterparts (i.e., first division) which may have affected the results. It is well established that younger players may have a greater room of improvement regarding their neuromuscular characteristics (Rinaldo et al., 2020). Furthermore, all jump tests were performed at the beginning of the preparation period (i.e., after reduced training loads); therefore, it cannot be confirmed whether the present findings would persist during the in-season, when these variables are more specifically trained. In this regard, future studies should further focus on this topic, analyzing the fluctuation of phase-specific CMJ metrics across different time-points during the entire basketball season (i.e., in and off season). Also, this study only analyzed variables related to the CMJ and it would be interesting to conduct a more specific analysis in other types of jumps as well (e.g., a squat jump, a reactive jump). Finally, these results were obtained in a sample of basketball players competing in Spain and generalizations to other competitions in other countries should be made with caution.

## Conclusions

This study compared a myriad of eccentric and concentric phase-specific CMJ force-time metrics across competitive levels and playing positions in male basketball players. Jump height and only four concentric variables (i.e., absolute peak force, absolute and relative peak power, and peak velocity) were observed to discriminate between competitive levels, with professional players outperforming semi-professional players. Conversely, players from different competitive levels displayed similar eccentric phase characteristics. Regarding playing positions, metrics related with both the eccentric and concentric phases were found to have a discriminative value. Overall, centers presented longer duration of eccentric deceleration and concentric phases, and greater output in absolute terms (e.g., eccentric absolute peak force, concentric absolute peak force and power). In contrast, guards tended to display superior performance in relative terms (e.g., concentric relative peak force). From an applied standpoint, practitioners are recommended to conduct an in-depth CMJ kinematic and kinetic analysis to better profile their players. Moreover, based on the present findings, it appears that concentric capabilities are determinant to compete at the highest level and that the capacity to produce absolute force and power seems to be particularly important in centers compared to other playing positions.
